# Co-Housing Rodents with Different Coat Colours as a Simple, Non-Invasive Means of Individual Identification: Validating Mixed-Strain Housing for C57BL/6 and DBA/2 Mice

**DOI:** 10.1371/journal.pone.0077541

**Published:** 2013-10-28

**Authors:** Michael Walker, Carole Fureix, Rupert Palme, Georgia Mason

**Affiliations:** 1 Animal and Poultry Science, University of Guelph, Guelph, Ontario, Canada; 2 University of Veterinary Medicine, Department of Biomedical, Sciences/Biochemistry, Vienna, Austria; Université de Bordeaux and Centre National de la Recherche Scientifique, France

## Abstract

Standard practice typically requires the marking of laboratory mice so that they can be individually identified. However, many of the common methods compromise the welfare of the individuals being marked (as well as requiring time, effort, and/or resources on the part of researchers and technicians). Mixing strains of different colour within a cage would allow them to be readily visually identifiable, negating the need for more invasive marking techniques. Here we assess the impact that mixed strain housing has on the phenotypes of female C57BL/6 (black) and DBA/2 (brown) mice, and on the variability in the data obtained from them. Mice were housed in either mixed strain or single strain pairs for 19 weeks, and their phenotypes then assessed using 23 different behavioural, morphological, haematological and physiological measures widely used in research and/or important for assessing mouse welfare. No negative effects of mixed strain housing could be found on the phenotypes of either strain, including variables relevant to welfare. Differences and similarities between the two strains were almost all as expected from previously published studies, and none were affected by whether mice were housed in mixed- or single-strain pairs. Only one significant main effect of housing type was detected: mixed strain pairs had smaller red blood cell distribution widths, a measure suggesting better health (findings that now need replicating in case they were Type 1 errors resulting from our multiplicity of tests). Furthermore, mixed strain housing did not increase the variation in data obtained from the mice: the standard errors for all variables were essentially identical between the two housing conditions. Mixed strain housing also made animals very easy to distinguish while in the home cage. Female DBA/2 and C57BL/6 mice can thus be housed in mixed strain pairs for identification purposes, with no apparent negative effects on their welfare or the data they generate. This suggests that there is much value in exploring other combinations of strains.

## Introduction

Individual identification provides the only link between a subject and the data collected from it. Many research paradigms and experiments therefore require the individual marking of laboratory rodents. Three broad methods are common: temporary markings (e.g. tail marking with a marker pen [1] or shaving a patch of hair [2]), permanent mutilations (e.g. ear notching [3] or toe clipping [4]), or the addition of permanent identification tags (e.g. tattooing [5] or micro-chipping [6]). Methods are constantly being refined and improved (e.g. [7]). Nevertheless, as we review below, all common marking methods have the potential to negatively impact animal welfare or influence the results obtained from them; they may also be laborious and/or costly for researchers.

Temporary markings, for example, often need to be reapplied at regular intervals (e.g. [1]), especially in mice [8], which is time-consuming. Human handling and restraint are also aversive and stressful to mice [9]–[11], as is the scent of marker pen to rats [1]. Furthermore, rats tail-marked with ink show altered behaviour in standardized tests (being more likely to enter, and spend more time in, the open arms of an elevated plus maze [1]). Turning to mutilations, ear notching without analgesia causes acute pain, as evidenced by a short term sympathetic stress response (assessed via increases in blood pressure [7]) and an increased number of audible vocalizations compared with sham treated control mice [12] (audible vocalizations are an established indicator of pain in rodents [13]). The toe clipping of neonatal mice (∼5–7 days old) does not appear to induce a stress response any more than regular handling, in contrast, nor have any negative long-term consequences on health or performance [4], [14]. However, some caution is needed here: there is a current lack of knowledge about the perception of pain in young rodents [15], and objectively assessing low-moderate pain in mice is also recognised as difficult [4], [16]. Furthermore, evidence from rats indicates that toe-clipping can impair later performance in certain behavioural tasks, such as the grip suspension test or a swimming task [5]. Toe-clips and ear-notches may also be hard for researchers to detect without very close proximity or handling, especially in animals within their home cages and/or under red light, in turn raising dangers of observer effects and making these marks inappropriate for identification in video recordings [8]. The last set of techniques is similarly invasive, but involves permanent identification tags such as tattoos and microchips. These methods require specialized equipment and some technical skill to administer. Traditional ear tattoo methods caused a significant acute increase in heart rate and blood pressure in rats (comparable to ear notching) [7], although apparently no long-term effects on growth, behaviour, or sensory-motor function [5], [14], [17]. Microchips are generally injected into the subcutaneous region of the dorsal surface of the rodent, sometimes with anaesthesia (e.g. [6], [18]), sometimes without (e.g. [19]). Microchips can be extremely valuable when used with technologies allowing automatic collection of behavioural and physiological data (e.g. [6], [20]), although they are obviously not appropriate when continuous visual/video monitoring is needed, because not detectable without a chip-reader. In terms of animal welfare, injection of the device is likely to be painful if conducted without anaesthesia (e.g. [19]). Furthermore, microchips have been implicated in tumour development [21]–[23]. These have only been found in older animals in long-term studies, and typically the incidence rate is low (1–4%); still, because the prognosis for animals with foreign body tumours is typically poor [24], this raises welfare concerns for these older subjects, as well as suggesting that microchips may be inappropriate for long-term or oncological studies. Finally, the Federation of European Laboratory Animal Science Associations (FELASA) has recently published a comprehensive overview of the protocols and procedures associated with all of the above identification methods [25]. In it, they identify all permanent marking techniques, from mutilations to implants and tattoos, as painful upon application (unless analgesics are used), and thus potentially a welfare concern.

Here we propose a new approach that would eliminate the need to mark individual animals: mixing visually distinctive strains within cages. In mice, for example, there are hundreds of strains, many of which can be readily visually distinguished. Coat pigmentation for instance, varies greatly as a function of genetic mutation [26]. Therefore, if mice from differentially pigmented strains were housed together, they could be distinguished as individuals. This would obviate needs for technical help in marking or specialized equipment; eliminate concerns about pain or stress resulting from marking practices; and allow great ease of identification from a distance, within the home cage, under red light, in video recordings, and even by many video tracking systems (e.g. Noldus EthoVision® XT) if appropriately contrasting backgrounds are used. In addition, using multiple strains of mice increases systematic variation within animal experiments (compared to experiments that only use a single strain), which will in turn lead to greater reproducibility and external validity of results [27]. However, our proposed novel mixed strain approach would only be ethically acceptable if it can be shown not to cause new welfare concerns; and only scientifically acceptable if it does not alter animals’ previously well-characterized phenotypes (e.g. [28]) or increase the variance of measured variables (so making it harder to detect significant effects) [29]. Therefore, in this preliminary study of two common strains we tested two hypotheses: that mixed strain housing affects the phenotypes of mice (including states related to welfare), and that mixed strain housing increases the variance in data obtained from the animals. We housed C57BL/6 (black) and DBA/2 (brown) females in either single or mixed strain pairs between 3 weeks (weaning age) and 22 weeks (when mice are well into adulthood), and took a total of 23 behavioural, physiological, morphological, and haematological measures.

## Methods

### Ethical Note

All procedures listed here were approved by the University of Guelph Animal Care Committee (Animal Utilization Protocol number: 1398) and comply with the Canadian Council on Animal Care guidelines.

### Animals & Housing

31 female, non-related, DBA/2 and 31 female, non-related, C57BL/6 mice were purchased from Charles River Labs at three weeks of age. We chose these inbred strains, not just for their different coat colours, but also because they are both widely used, comparable in body weight [30], and similarly sociable [28]. We used females because they are commonly group-housed [31], necessitating individual identification, and because females make up a large proportion (approximately 70%) of the inbred mice sold by Charles River Laboratories (personal communication).

Upon arrival, mice were randomly divided up into either same strain or mixed strain pairs. The day after arrival, all mice were given carprofen in their water supply, and the next day, once analgised [32], one mouse in each single strain cage was ear notched. Carprofen was continued for a day afterwards. Due to a few malocclusion cases, the final experimental setup comprised: 9 DBA/2 pairs, 8 C57BL/6 pairs, and 11 mixed strain pairs (total n = 56). Mice were all housed in conventional polysulfone plastic ‘shoebox’ cages (12 *H*cm×27 *L*cm×16 *W*cm; Allentown, Inc.) on shelves in a room kept at 21±1°C and 48% relative humidity and was on a 12-hour reverse light schedule (lights out at 10 am). The cages were arranged systematically along the shelves in a rotating pattern between the three different cage setups, so that all cage-types were evenly represented on each of three shelves. The cages were furnished with corncob bedding, Shepherd Shack Enviro-dry© nesting material, a UDEL polysulfone plastic mouse house shelter and *ad lib.* food and water. The cages were completely cleaned once a week.

### Preliminary Behavioural Data Collection

After six weeks of differential housing, preliminary home cage observations and behavioural tests were conducted for two weeks in order to ensure behavioural compatibility between cage mates, and also validate and finalize all testing protocols. During this time, it was determined that some mice were more active in the early part of the day and others during the later part, shaping our final test schedule (see below). Behavioural observers (MW & CF) were trained, and their independently-collected data were then compared for intra- and inter-observer reliability for all behavioural observations (p always <0.05 for all variables by the end of training). For home cage data, 16 hours of observation over two days were also ascertained to be sufficient to produce reliable, consistent results. No aggression was observed between cage mates, and so they were left in their current pairs for an additional seven weeks before the final data collection phase. Data were collected in the order below, and no data were ever collected on a cage-cleaning day.

### Home Cage Time Budgets During the Active Phase

Home cage observations were conducted in two four-hour blocks per day (12 pm–4 pm; 5 pm–9 pm) during the dark period, for two days. On Day 1, MW observed mice in the early block and CF observed them in the late block, this being reversed on Day 2. The silent observer recorded them every 12 minutes during the block, using a mixture of focal and scan sampling [33], and following a previously determined, well-validated, ethogram (see [34] for details). For analysis, behaviour types were pooled into three categories: normal activity (e.g. locomotion, grooming, eating/drinking), inactivity (e.g. standing still, sleeping), and stereotypic behaviour (e.g. repetitive route tracing, patterned climbing, involving elements repeated three or more times). Behaviours that did not fall within these categories, such as borderline stereotypies (i.e. only two repetitions of a behavioural pattern), were scored as ‘ambiguous’. These behavioural variables were selected to allow comparison with published strain typical values ( [35], [36]) and for their use in assessing mouse welfare [37].

#### Behavioural tests

For all tests, any test that required more than one trial was conducted at an early time one day (12 pm), and a later time on the next day (5 pm), so that all subjects would be assessed during one of their active times (see Preliminary behavioural data collection). Behavioural tests began after 13 weeks of differential housing and continued for three weeks, with no more than one test/trial being performed per day. Each test was selected to allow comparison with published strain typical values (e.g. [38]) and for their potential value in mouse welfare assessment (e.g. [39]).

### Sucrose Consumption Test

Lower levels of sucrose consumption indicate increased anhedonia (e.g. [40]). This is usually assessed via ingestion of sucrose solution, but the use of solid sucrose is a validated alternative [41]. To collect individual data on sucrose consumption, mice were placed individually for 30 minutes in wire mesh compartments (0.64 cm×0.64 cm mesh) that fitted inside their home cage, and contained a sugar lump, along with a normal food pellet (as an experimental control for feeding motivation). These compartments were designed to separate the mice physically while still allowing them visual and olfactory contact with each other. This was conducted for five consecutive days pre-test, to habituate mice so that stress and hyponeophagia responses would be minimized. Two test trials were then conducted, one on each of two consecutive days. The sugar lump was weighed before and after each of these tests, and an average taken to quantify sugar consumption per mouse. Mice were weighed at the end of the second trial so that body weight could be added to the statistical model as a blocking factor for the analysis. Finally, to check that the mesh compartments did not affect sucrose consumption, two pre-weighed sugar cubes were placed in the home cage for 30 minutes, on two consecutive days, with both mice thus allowed equal access (*cf.* e.g. [2]). These consumption values were regressed against the average values for both cage mates in the trials with the mesh compartments. Sugar consumption correlated strongly between the two types of test (R^2^ = 0.43, F_1,22_ = 14.09, p = 0.001), thus validating our new technique.

### Novel Object Test

Long latencies to make contact with a novel object are typically interpreted as reflecting higher levels of anxiety or neophobia (e.g. [42]). To assess this, we used a previously determined protocol [2], involving exposing mice to a novel object in their home cage by inserting it through the cage lid. Two trials were conducted, one at 12 pm (using a standard wooden popsicle stick) and one the following day at 5 pm (using a white plastic fork). After an object had been used once, it was discarded, each cage always being tested with a new item, so that no odour cues were left on the object between cages. The maximum allowed duration was five minutes; any mouse making no contact at all was given the maximum score (300 seconds).

### Startle Response Test

Large responses to sudden auditory tones reflect more anxious phenotypes [43]. Acoustic startle responses were assessed using four Kinder Scientific startle boxes and Startle Monitor software for analysis. The four startle boxes were calibrated prior to use using the protocol provided by the manufacturer. In batches of four, mice were each placed individually in one box such that they could move around but could not rear up, and were allowed to habituate to the box for 6 minutes (50 dB white background noise). At the 6-minute mark, a loud (115 dB for 40 ms) auditory tone was played in all four boxes simultaneously. The force generated by each mouse immediately prior to the tone was recorded (to account for the body weight of the mouse), as was the force generated by the mouse over the duration of the tone. The startle response was calculated as the peak force minus the initial force.

### Physiological, Haematological and Morphological Data

#### Baseline levels of faecal corticosterone metabolites

Faeces were collected from each mouse during the startle response test and then during a half hour period of isolation three days later. Rodents tend to defecate in response to stressors [44], and because corticosterone metabolites gradually accumulate in the faeces after a delay of several hours (reviewed [45]), this method is a good way to collect samples that reflect baseline levels of circulating corticosterone. The two samples were pooled per mouse and then frozen at −20°C until processed as follows: each sample was homogenized and an aliquot of 0.05 g was shaken with 1 ml of 80% methanol; after centrifugation, an aliquot of the supernatant was diluted (1∶20) with assay buffer and frozen at −20°C until analysis. A 5α-pregnane-3β,11β,21-triol-20-one EIA, which has proven well suited to assess corticosterone metabolites in mouse faeces, was used for analysis (for details see [46]; for validation for mice, see [47]). Nine mice did not produce enough faeces for a complete assay, so were not counted in the analysis.

### Body Condition

Mice were weighed immediately prior to euthanasia so that we could use body weight as a dependent variable, and also include it as a blocking factor in the model for spleen weight. All mice were euthanized three weeks following the end of behavioural testing, and a gross examination of body condition was done, specifically looking for bite marks/wounds and evidence of barbering (an abnormal behaviour where a mouse will pluck the whiskers or body hair from itself or a cage mate [48]).

### Post Mortem Measures

Euthanasia was conducted by cervical dislocation after 19 weeks of differential housing, and was performed by a trained technician. Immediately following death, a blood sample was taken via cardiac puncture. A small sample of blood was used to determine blood glucose, using a Contour® blood glucose meter; the rest of the sample was put into a heparinized tube (∼50 µL). After this, the mouse was dissected and the spleen was removed and weighed. Spleen mass is likely to reflect immune status in mammals (larger spleens suggest higher immune-competence) [49], and also possibly inherently differs between C57BL/6 and DBA/2 mice [50]. Heparinized blood samples were sent to the University of Guelph Animal Health Laboratory for a Complete Blood Count analysis. Ten samples were lost due to clotting prior to analysis (six “single” DBA/2; two “mixed” DBA/2; two “single” C57BL/6).

### Statistical Analysis

All analyses were conducted in JMP® 10. General linear models (GLMs) were used to test all hypotheses (except where otherwise indicated), and to run the behavioural consistency checks mentioned in the Methods. Originally, ear notching (Y/N) was included in all models, but this was never a significant effect (p always >0.10) and so was removed. The GLM used for each dependent variable was similar:
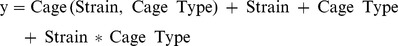



Cage is a blocking factor in order to avoid pseudoreplication because mice housed in the same cage are non-independent (see [51], [52]), and was set as a random effect [53]. Strain and Cage Type are both nested within Cage (Cage Type being either single or mixed strain). In certain cases, additional terms were added to reflect other variables considered necessary as controls in specific analyses (e.g. body weight in the sucrose consumption analysis). Type 3 sums of squares were used except when there was a continuous variable in the model (causing non-orthogonality), in which case Type 1 sums of squares were used, with each term of interest being placed last in the model in turn [29]. Data were transformed where necessary to fit the parametric assumptions of GLMs. If mixed strain housing alters phenotypes, Cage Type would have significant effects; and if mixed strain housing altered the magnitude of strain differences (a more important concern), Cage Type*Strain would be significant. Although a total of 69 p-values were generated during hypothesis testing, we did not control for multiple testing; this was to increase our ability to detect any effects of mixing strains, although it potentially made us vulnerable to Type 1 errors (see Discussion).

To investigate the impact of mixed strain housing on the variability of measures, we ran three additional tests on the standard errors of the dependent variables. 23 standard error values for each housing type were used in a GLM to test for differences between Cage Types, blocking by strain; and also to assess their co-variance. Since the slope of relationship between the two sets of values did not vary with Strain (see Results), both strains were pooled to enable a linear regression in which we tested the null hypothesis that the slope of the line was 1.

## Results

### Home Cage Time Budgets

Behavioural consistency between days proved to be very high (inactivity: F_1,52_ = 33.12, p<0.001; normal activity: F_1,52_ = 21.59, p<0.001; stereotypic behaviour: F_1,52_ = 44.57, p<0.001). Because ambiguous behaviours were rare (<5% of observations), they were not included in any analyses. The two strains differed in time budgets, with DBA/2 s being more stereotypic, and thence less inactive as well as spending less time in normal activity (Table 1). However, the magnitude and direction of strain differences were unaffected by mixed strain housing: Cage Type*Strain never approached significance (Table 2). The one possible main effect of Cage Type on both strains was a trend for mice in mixed strain cages to be less stereotypic than their same-strain peers in single strain cages (Table 3). Aggressive interactions were never observed (and nor did the animal care technician ever report any behavioural issues over the duration of the experiment).

**Table 1:** Descriptive and test statistics for behavioural, morphological, and physiological data for each Strain (C57BL/6 or DBA/2).

Variable	Strain	Mean (95% CI)	Strain main effect statistics	p
**Inactivity (% of scans)**	C57BL/6	17.7 (12.2–24.8)	F_1,37_ = 28.35	**<0.001**
	DBA/2	4.2 (2.8–6.2)		
**Normal Activity (% of scans)**	C57BL/6	73.4 (68.5–77.8)	F_1,39_ = 8.15	**0.007**
	DBA/2	63.3 (57.7–68.5)		
**Stereotypy (% of scans)**	C57BL/6	4.9 (3.2–8.9)	F_1,40_ = 43.37	**<0.001**
	DBA/2	27.8 (20.0–37.2)		
**Novel Object (s)**	C57BL/6	42.7 (30.4–59.8)	F_1,42_ = 19.55	**<0.001**
	DBA/2	15.1 (11.0–21.0)		
**Sucrose Consumption (g)**	C57BL/6	0.083 (0.064–0.11)	F_1,39_ = 1.61	0.213
	DBA/2	0.067 (0.052–0.085)		
**Startle Response (N)**	C57BL/6	0.144 (0.078–0.16)	F_1,47_ = 12.00	**0.001**
	DBA/2	0.044 (0.031–0.064)		
**Body Weight (g)**	C57BL/6	21.9 (21.40–22.5)	F_1,42_ = 0.033	0.857
	DBA/2	22.0 (21.5–22.5)		
**Spleen Weight (g)**	C57BL/6	0.081 (0.078–0.085)	F_1,29_ = 1.81	0.190
	DBA/2	0.083 (0.080–0.086)		
**Blood Glucose (mmol/L)**	C57BL/6	8.5 (7.7–9.4)	F_1,37_ = 3.70	0.062
	DBA/2	7.5 (6.7–8.3)		
**FCM** * **(ng/0.05** **g of faeces)**	C57BL/6	53.7 (43.4–66.5)	F_1,30_ = 11.50	**0.002**
	DBA/2	85.9 (71.4–103.3)		

* Faecal Corticosterone Metabolites.

**Table 2:** Descriptive and test statistics for behavioural, morphological, and physiological data for each Strain (C57BL/6 or DBA/2), split by Cage Type (single strain or mixed strain).

Variable	Strain & Cage Type	Mean (95% CI)	Strain*Cage Typeinteraction statistics	p
**Inactivity (% of scans)**	C57BL/6 Mixed	20.2 (11.9–32.2)	F_1,37_ = 1.37	0.250
	C57BL/6 Single	15.3 (9.0–24.9)		
	DBA/2 Mixed	3.5 (1.9–6.4)		
	DBA/2 Single	5.0 (2.9–8.5)		
**Normal Activity (% of scans)**	C57BL/6 Mixed	75.4 (67.9–81.6)	F_1,39_ = 0.15	0.703
	C57BL/6 Single	71.3 (64.5–77.3)		
	DBA/2 Mixed	67.1 (58.5–74.6)		
	DBA/2 Single	59.3 (52.0–66.2)		
**Stereotypy (% of scans)**	C57BL/6 Mixed	3.1 (1.6–5.9)	F_1,40_ = 1.25	0.270
	C57BL/6 Single	7.6 (4.3–12.9)		
	DBA/2 Mixed	25.3 (14.8–39.7)		
	DBA/2 Single	30.4 (20.0–43.4)		
**Novel Object (s)**	C57BL/6 Mixed	41.3 (24.9–68.6)	F_1,42_ = 0.0002	0.988
	C57BL/6 Single	44.0 (27.9–69.2)		
	DBA/2 Mixed	14.7 (8.9–24.4)		
	DBA/2 Single	15.5 (10.1–23.9)		
**Sucrose Consumption (g)**	C57BL/6 Mixed	0.09 (0.06–0.13)	F_1,40_ = 0.054	0.809
	C57BL/6 Single	0.08 (0.05–0.11)		
	DBA/2 Mixed	0.08 (0.05–0.11)		
	DBA/2 Single	0.06 (0.04–0.08)		
**Startle Response (N)**	C57BL/6 Mixed	0.12 (0.07–0.21)	F_1,47_ = 0.071	0.791
	C57BL/6 Single	0.11 (0.07–0.18)		
	DBA/2 Mixed	0.05 (0.03–0.09)		
	DBA/2 Single	0.04 (0.03–0.06)		
**Body Weight (g)**	C57BL/6 Mixed	21.5 (20.6–22.3)	F_1,42_ = 0.82	0.370
	C57BL/6 Single	22.4 (21.7–23.1)		
	DBA/2 Mixed	21.9 (21.0–22.7)		
	DBA/2 Single	22.2 (21.5–22.8)		
**Spleen Weight (g)**	C57BL/6 Mixed	0.082 (0.077–0.088)	F_1,42_ = 2.11	0.154
	C57BL/6 Single	0.080 (0.076–0.085)		
	DBA/2 Mixed	0.081 (0.076–0.086)		
	DBA/2 Single	0.085 (0.081–0.090)		
**Blood Glucose (mmol/L)**	C57BL/6 Mixed	8.6 (7.4–9.7)	F_1,37_ = 0.092	0.763
	C57BL/6 Single	8.5 (7.4–9.7)		
	DBA/2 Mixed	7.7 (6.5–8.8)		
	DBA/2 Single	7.3 (6.2–8.4)		
**FCM** * **(ng/0.05 g of faeces)**	C57BL/6 Mixed	48.6 (35.0–67.4)	F_1,30_ = 0.99	0.328
	C57BL/6 Single	59.3 (44.9–78.3)		
	DBA/2 Mixed	89.2 (67.9–117.2)		
	DBA/2 Single	82.6 (64.2–106.4)		

* Faecal Corticosterone Metabolites.

**Table 3:** Descriptive and test statistics for behavioural, morphological, and physiological data for each Cage Type (single strain or mixed strain).

Variable	Cage Type	Mean (95% CI)	Cage Type main effect statistics	p
**Inactivity (% of scans)**	Mixed	8.7 (5.8–13.0)	F_1,37_ = 0.003	0.959
	Single	8.8 (6.0–12.8)		
**Normal Activity (% of scans)**	Mixed	71.4 (65.8–76.5)	F_1,39_ = 2.74	0.106
	Single	65.5 (60.5–70.4)		
**Stereotypy (% of scans)**	Mixed	10.5 (6.1–14.3)	F_1,40_ = 3.83	0.057
	Single	15.9 (11.2–22.2)		
**Novel Object (s)**	Mixed	24.7 (17.3–35.3)	F_1,42_ = 0.062	0.805
	Single	26.2 (19.1–35.7)		
**Sucrose Consumption (g)**	Mixed	0.084 (0.064–0.11)	F_1,39_ = 1.75	0.193
	Single	0.066 (0.052–0.084)		
**Startle Response (N)**	Mixed	0.077 (0.053–0.11)	F_1,32_ = 0.38	0.541
	Single	0.066 (0.047–0.092)		
**Body Weight (g)**	Mixed	21.7 (21.0–22.2)	F_1,42_ = 2.86	0.098
	Single	22.3 (21.8–22.8)		
**Spleen Weight (g)**	Mixed	0.082 (0.078–0.085)	F_1,42_ = 0.25	0.617
	Single	0.083 (0.080–0.086)		
**Blood Glucose (mmol/L)**	Mixed	8.1 (7.3–8.9)	F_1,37_ = 0.11	0.738
	Single	7.9 (7.1–8.7)		
**FCM** * **(ng/0.05** **g of faeces)**	Mixed	65.9 (53.3–81.5)	F_1,30_ = 0.20	0.662
	Single	70.0 (58.0–84.5)		

* Faecal Corticosterone Metabolites.

### Behavioural Tests

Again, marked strain differences were evident, at least in the two tests related to fear and anxiety (novel object test and startle response test); DBA/2 mice had shorter latencies to touch the novel objects and were less reactive in the startle response test (Table 1). However, mixed strain housing had no influence on results (Table 2). Anhedonia was unaffected by Strain, Cage Type, or its interaction. This result was consistent whether or not ‘body weight’ was included in the model (not in practice a predictor of sugar consumption [F_1,50_ = 0.026, p = 0.873]), so we kept it in the model as it best tests the hypothesis, taking potential biological confounds into account.

### Physiological, Haematological, and Morphological Variables

Strain affected hematocrit, haemoglobin, and mean corpuscular volume (Table 4), and levels of faecal corticosterone metabolites (FCM; Table 1); strain also showed a trend to affect blood glucose (Table 1). However, like the behavioural measures, these strain effects did not interact with Cage Type (Tables 2 & 5). Cage Type had one significant main effect; red blood cell distribution width was significantly higher in single strain pairs (Table 6). Cage Type showed weak trends to affect basophil counts, single-strain mice having lower levels, and to affect body weight, with mice in single strain pairs being slightly heavier (Table 3); however there were no interactions between these measures and Strain. The blood glucose result was unchanged by the inclusion of ‘time since food removal’ (a significant influence on glucose [F_1,35_ = 6.98, p = 0.012]), and spleen weight was unchanged by the inclusion of ‘body weight’ (a significant predictor of spleen weight [F_1,34_ = 48.84, p<0.001]), so we left them in the model to best test our hypotheses by taking biological confounds into account. No evidence of bite marks, wounds, or barbering was found *post mortem*.

**Table 4:** Descriptive and test statistics for haematological data for each Strain (C57BL/6 or DBA/2).

Variable	Strain	Mean (95% CI)	Strain main effect statistics	p
**White Blood Cell Count (×10^9^/L)**	C57BL/6	2.5 (1.2–3.3)	F_1,30_ = 0.77	0.387
	DBA/2	2.1 (1.6–2.81)		
**Red Blood Cell Count (×10^12^/L)**	C57BL/6	9.5 (9.2–9.8)	F_1,34_ = 0.017	0.899
	DBA/2	9.5 (9.2–9.8)		
**Haemoglobin (g/L)**	C57BL/6	139.6 (135.9–143.4)	F_1,34_ = 6.94	**0.013**
	DBA/2	132.4 (128.3–136.5)		
**Hematocrit (L/L)**	C57BL/6	0.47 (0.43–0.49)	F_1,35_ = 5.23	**0.031**
	DBA/2	0.44 (0.42–0.46)		
**Mean Corpuscular Volume (fL)**	C57BL/6	49.6 (49.1–50.1)	F_1,33_ = 76.70	**<0.001**
	DBA/2	46.6 (46.0–47.1)		
**MCHC** * **(g/L)**	C57BL/6	297.8 (293.4–302.1)	F_1,30_ = 0.67	0.420
	DBA/2	300.3 (295.6–305.0)		
**RDW** § **(%)**	C57BL/6	12.7 (12.5–12.9)	F_1,34_ = 180.3	**<0.001**
	DBA/2	14.4 (14.2–14.5)		
**Mean Platelet Volume (fL)**	C57BL/6	14.4 (11.2–17.7)	F_1,33_ = 1.38	0.249
	DBA/2	17.2 (13.7–20.8)		
**Absolute Neutrophils (×10^9^/L)**	C57BL/6	0.29 (0.22–0.39)	F_1,31_ = 0.003	0.959
	DBA/2	0.28 (0.21–0.39)		
**Absolute Lymphocytes (×10^9^/L)**	C57BL/6	1.9 (1.4–2.6)	F_1,29_ = 0.51	0.482
	DBA/2	1.6 (1.1–2.2)		
**Absolute Eosinophils (×10^9^/L)**	C57BL/6	0.038 (0.029–0.049)	F_1,31_ = 10.32	**0.003**
	DBA/2	0.068 (0.052–0.089)		
**Absolute Monocytes(×10^9^/L)**	C57BL/6	0.069 (0.026–0.11)	F_1,27_ = 0.002	0.969
	DBA/2	0.087 (0.041–0.13)		
**Absolute Basophils (×10^9^/L)**	C57BL/6	0.034 (0.024–0.047)	F_1,27_ = 1.28	0.246
	DBA/2	0.025 (0.018–0.036)		

* Mean Corpuscular Haemoglobin Concentration.

§ Red Blood Cell Distribution Width.

**Table 5:** Descriptive and test statistics for haematological data for each Strain (C57BL/6 or DBA/2) split by Cage Type (single strain or mixed strain).

Variable	Strain & Cage Type	Mean (95% CI)	Strain*Cage Typeinteraction statistics	p
**White Blood Cell Count (×10^9^/L)**	C57BL/6 Mixed	2.9 (2.0–4.2)	F_1,30_ = 1.26	0.271
	C57BL/6 Single	2.1 (1.4–3.2)		
	DBA/2 Mixed	2.0 (1.3–3.0)		
	DBA/2 Single	2.2 (1.4–3.4)		
**Red Blood Cell Count (×10^12^/L)**	C57BL/6 Mixed	9.6 (9.1–10.0)	F_1,34_ = 0.38	0.542
	C57BL/6 Single	9.4 (9.0–9.8)		
	DBA/2 Mixed	9.7 (9.2–10.2)		
	DBA/2 Single	9.3 (8.9–9.7)		
**Haemoglobin (g/L)**	C57BL/6 Mixed	140.6 (135.1–146.2)	F_1,33_ = 0.39	0.539
	C57BL/6 Single	138.6 (133.5–143.8)		
	DBA/2 Mixed	135.1 (129.0–141.2)		
	DBA/2 Single	129.7 (124.1–135.5)		
**Hematocrit (L/L)**	C57BL/6 Mixed	0.47 (0.44–0.50)	F_1,35_ = 0.823	0.371
	C57BL/6 Single	0.47 (0.45–0.49)		
	DBA/2 Mixed	0.45 (0.43–0.48)		
	DBA/2 Single	0.43 (0.41–0.45)		
**Mean Corpuscular Volume (fL)**	C57BL/6 Mixed	49.0 (48.3–49.7)	F_1,33_ = 2.83	0.102
	C57BL/6 Single	50.2 (49.5–50.8)		
	DBA/2 Mixed	46.6 (45.8–47.4)		
	DBA/2 Single	46.6 (45.9–47.3)		
**MCHC** * **(g/L)**	C57BL/6 Mixed	299.9 (293.7–306.4)	F_1,30_ = 1.31	0.262
	C57BL/6 Single	295.6 (289.6–301.6)		
	DBA/2 Mixed	298.9 (291.8–306.0)		
	DBA/2 Single	301.7 (295.3–308.2)		
**RDW** § **(%)**	C57BL/6 Mixed	12.6 (12.4–12.9)	F_1,34_ = 2.01	0.166
	C57BL/6 Single	12.8 (12.6–13.0)		
	DBA/2 Mixed	14.1 (13.8–14.4)		
	DBA/2 Single	14.6 (14.4–14.9)		
**Mean Platelet Volume (fL)**	C57BL/6 Mixed	14.7 (10.0–19.4)	F_1,33_ = 0.16	0.688
	C57BL/6 Single	14.2 (9.6–18.7)		
	DBA/2 Mixed	16.5 (11.3–21.8)		
	DBA/2 Single	17.9 (13.0–22.8)		
**Absolute Neutrophils (×10^9^/L)**	C57BL/6 Mixed	0.27 (0.18–0.41)	F_1,31_ = 0.091	0.765
	C57BL/6 Single	0.31 (0.21–0.46)		
	DBA/2 Mixed	0.28 (0.18–0.46)		
	DBA/2 Single	0.29 (0.18–0.44)		
**Absolute Lymphocytes (×10^9^/L)**	C57BL/6 Mixed	2.29 (1.51–3.48)	F_1,29_ = 1.38	0.250
	C57BL/6 Single	1.54 (0.99–2.38)		
	DBA/2 Mixed	1.52 (0.96–2.41)		
	DBA/2 Single	1.70 (1.06–2.72)		
**Absolute Eosinophils (×10^9^/L)**	C57BL/6 Mixed	0.037 (0.026–0.053)	F_1,31_ = 0.0004	0.984
	C57BL/6 Single	0.039 (0.027–0.056)		
	DBA/2 Mixed	0.065 (0.045–0.095)		
	DBA/2 Single	0.070 (0.048–0.103)		
**Absolute Monocytes (×10^9^/L)**	C57BL/6 Mixed	0.059 (0.00–0.119)	F_1,27_ = 0.008	0.929
	C57BL/6 Single	0.079 (0.017–0.141)		
	DBA/2 Mixed	0.086 (0.023–0.150)		
	DBA/2 Single	0.087 (0.021–0.153)		
**Absolute Basophils (×10^9^/L)**	C57BL/6 Mixed	0.049 (0.030–0.079)	F_1,28_ = 1.40	0.246
	C57BL/6 Single	0.023 (0.014–0.038)		
	DBA/2 Mixed	0.028 (0.017–0.045)		
	DBA/2 Single	0.023 (0.014–0.040)		

* Mean Corpuscular Haemoglobin Concentration.

§ Red Blood Cell Distribution Width.

**Table 6:** Descriptive and test statistics for haematological data for each Cage Type (single strain or mixed strain).

Variable	Cage Type	Mean (95% CI)	Cage Type main effect statistics	p
**White Blood Cell Count (×10^9^/L)**	Mixed	2.4 (1.8–3.2)	F_1,30_ = 0.30	0.587
	Single	2.2 (1.6–2.9)		
**Red Blood Cell Count (×10^12^/L)**	Mixed	9.7 (9.3–10.0)	F_1,34_ = 2.18	0.149
	Single	9.3 (9.0–9.6)		
**Haemoglobin (g/L)**	Mixed	137.9 (133.7–142.0)	F_1,34_ = 1.81	0.187
	Single	134.2 (130.4–138.0)		
**Hematocrit (L/L)**	Mixed	0.46 (0.44–0.48)	F_1,35_ = 0.98	0.328
	Single	0.45 (0.43–0.47)		
**Mean Corpuscular Volume (fL)**	Mixed	47.8 (47.2–48.3)	F_1,33_ = 2.85	0.101
	Single	48.4 (47.9–48.8)		
**MCHC** * **(g/L)**	Mixed	299.4 (294.6–304.2)	F_1,30_ = 0.054	0.818
	Single	298.7 (294.3–303.1)		
**RDW** § **(%)**	Mixed	13.4 (13.2–13.5)	F_1,34_ = 8.77	**0.006**
	Single	13.7 (13.6–13.9)		
**Mean Platelet Volume (fL)**	Mixed	15.6 (12.1–19.1)	F_1,33_ = 0.031	0.862
	Single	16.0 (12.7–19.4)		
**Absolute Neutrophils (×10^9^/L)**	Mixed	0.28 (0.20–0.38)	F_1,31_ = 0.11	0.743
	Single	0.30 (0.22–0.40)		
**Absolute Lymphocytes (×10^9^/L)**	Mixed	1.9 (1.4–2.6)	F_1,29_ = 0.44	0.513
	Single	1.6 (1.2–2.2)		
**Absolute Eosinophils (×10^9^/L)**	Mixed	0.049 (0.038–0.064)	F_1,31_ = 0.14	0.708
	Single	0.052 (0.040–0.068)		
**Absolute Monocytes(×10^9^/L)**	Mixed	0.073 (0.029–0.12)	F_1,27_ = 0.39	0.536
	Single	0.083 (0.038–0.13)		
**Absolute Basophils (×10^9^/L)**	Mixed	0.037 (0.026–0.052)	F_1,27_ = 3.80	0.061
	Single	0.023 (0.016–0.033)		

* Mean Corpuscular Haemoglobin Concentration.

§ Red Blood Cell Distribution Width.

### Effects of Mixed Strain Housing on Variance

**Figure 1:**
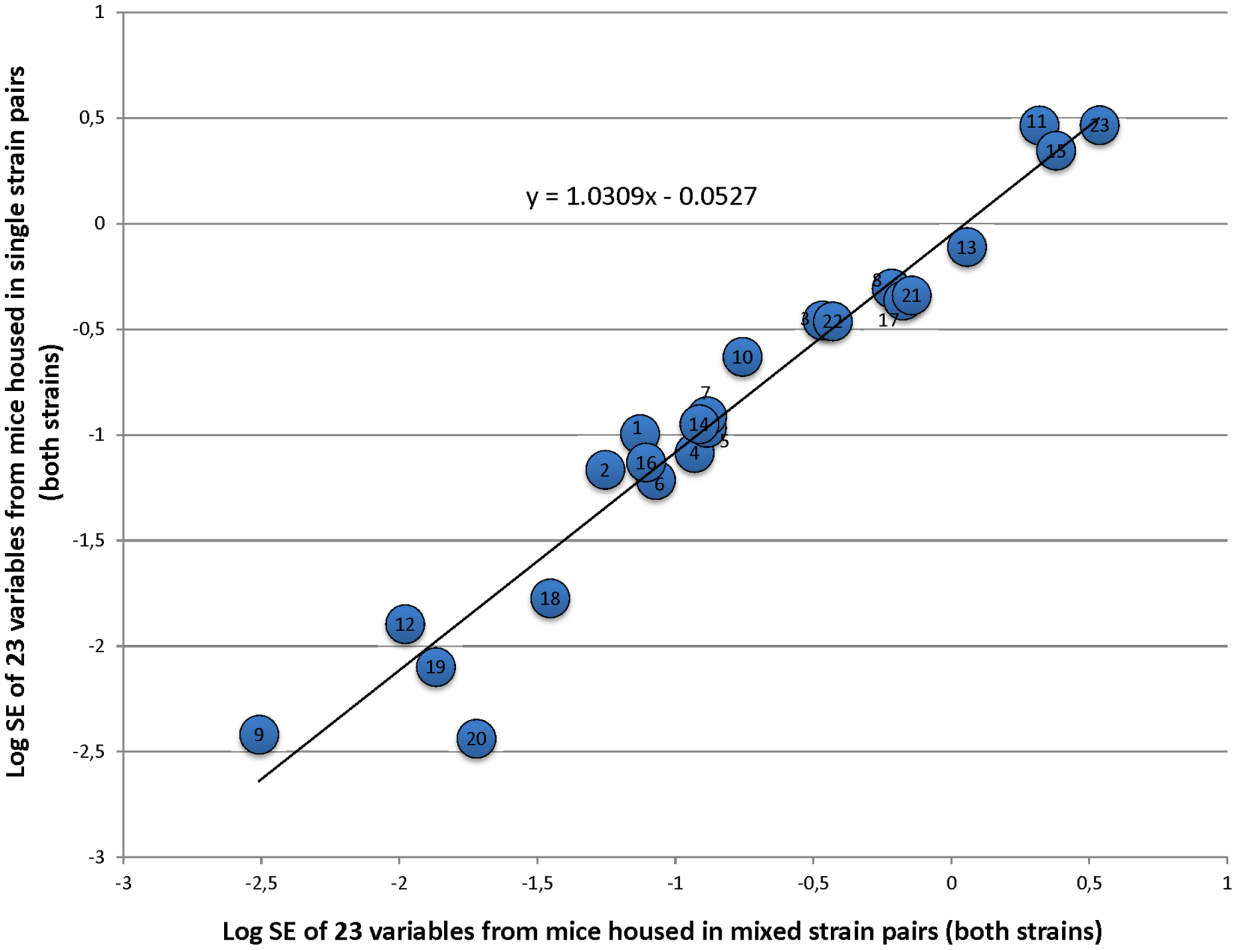
Relationship of the standard errors of 23 dependent variables between single- and mixed-strain housing. Each point represents the standard errors of one dependent variable labeled as follows: 1) Novel Object 2) Sucrose Consumption 3) Body Weight 4) Startle Response 5) Inactivity 6) Normal Activity 7) Stereotypy 8) Blood Glucose 9) Spleen Weight 10) Red Blood Cell Count 11) Haemoglobin 12) Hematocrit 13) Faecal Corticosterone Metabolites 14) Red Blood Cell Distribution Width 15) Mean Platelet Volume 16) Absolute Neutrophils 17) Absolute Lymphocytes 18) Absolute Monocytes 19) Absolute Eosinophils 20) Absolute Basophils 21) White Blood Cell Count 22) Mean Corpuscular Volume 23) Mean Corpuscular Haemoglobin Concentration. Data shown here have been log transformed (as analysed) to best show the linear relationship.

There were no significant differences in the variables’ standard errors between the two Cage Types (F_1,88_ = 0.11, p = 0.738). The standard errors co-varied closely between the two Cage Types (F_1,42_ = 641.6, p<0.001) and were not affected by Strain (F_1,42_ = 0.40, p = 0.53). Furthermore, the linear regression of one Cage Type against the other (Fig. 1) revealed that the slope of the relationship did not differ from one (F_1,21_ = 2.88, p = 0.104).

## Discussion

Several indicators were used to determine the impact of mixed strain housing on mouse welfare, namely stereotypic behaviours and barbering, anhedonia and anxiety/fear under test, faecal corticosterone metabolites (FCM), and body condition (including weight). In no case was any significantly affected by mixed strain housing. Two trend effects suggested mixed strain mice to be less stereotypic but have smaller body weights than their single strain peers (although because we did not correct for multiple comparisons these may be Type 1 errors, and so these results need replicating). Notably, there was a complete lack of aggressive interactions, barbering, and wounds indicating good behavioural compatibility between all cage mates, regardless of whether housed with a like strain. Thus overall, being in mixed strain C57BL-6-DBA/2 pairs did not compromise the welfare of our subjects.

Our second concern was that mixed strain housing might affect normal strain effects on phenotype: thus expected differences between DBA/2 and C57BL/6 mice could be altered in magnitude or even direction by mixed strain housing. There was no evidence of this. Consequently looking first at the indicators that were used to evaluate welfare, stereotypic behaviours (e.g. route tracing) were performed more frequently by DBA/2 mice than C57BL/6 mice, as expected from previous studies [35]. DBA/2 mice were also bolder in the novel object tests and less reactive in the startle response tests, indicating lower levels of ‘trait’ anxiety (cf. ‘state’ anxiety) [54], [55], consistent with known strain differences in startle responses [56] as well as with data from open field tests measuring the same trait [57], [58]. Again this strain difference was similarly expressed in single- and mixed-strain pairs, as was a strain difference in FCM: DBA/2 mice had higher baseline FCM levels than C57BL/6 s, regardless of housing type, a result consistent with known strain differences in endocrine response to stressors such as restraint [59], [60]. Body weights in contrast did not differ between strains, regardless of how housed: this lack of strain effect was, again, an expected finding [30]. Finally, no effect of strain or its interaction with cage type was found on anhedonia either. Other studies had found significant strain differences between C57BL/6 and DBA/2 mice (e.g. [38]), but only in animals subjected to unpredictable chronic mild stress; in our housing conditions the lack of strain difference in this variable was therefore again an expected finding.

A further 17 other variables were quantified including: blood glucose, spleen weight, home cage activity and inactivity levels, and numerous haematological measures. Once again, no strain-by-cage type interactions were found: any strain differences detected were thus as expected, and all were stable across mixed- and single-strain housing. One such effect was a strong trend for C57BL/6 mice to have higher blood glucose (regardless of housing type): a strain difference consistent with published literature [61]. C57BL/6 mice also had higher haemoglobin and hematocrit levels, and higher mean corpuscular volume, but lower levels of eosinophils than DBA/2 mice, again regardless of housing type, and all as consistent with the strain differences reported in The Jackson Laboratory’s mouse phenome database [62]. One surprising finding was that spleen weight did not differ between these two strains (cf. [50]), although the direction of non-significant effect was in the predicted direction (with DBA/2 s having higher values). This could reflect low power, or instead that the previous findings from males [50] do not apply to females. A second surprise was the emergence of one main effect of cage type: mice housed in single strain pairs had significantly higher red blood cell distribution widths (RDW) compared to peers in mixed strain cages. RDW, a measure of the variation in red blood cell size, was found to be a significant predictor of all-cause mortality in a long term study on humans [63]. Like our stereotypy finding, this suggests that mixed-strain housing may have some benefits, although likewise it should be treated with caution until replicated (as a potential Type 1 error). Overall, the fact that there were no strain-by-cage type interactions for any of the 23 variables measured, and that many well-established strain differences were maintained in our mixed strain pairs, indicates that the mixed strain housing used here has no readily detectable effects on mouse phenotype.

Our third research question was whether this form of mixed-strain housing would adversely affect inter-individual variation, so potentially increasing the numbers of subjects needed to detect significant effects. We found no evidence that mixed-strain housing increases data variability: for all variables, the standard errors of data from mixed-strain-housed mice proved extremely similar to those from same-strain-housed animals. If data variance had been increased by mixed-strain housing, then using this paradigm would mean more animals would be needed in order to obtain the same degree of statistical power as single-strain housing: not cost-effective and a clear violation of the 3Rs [64]. However, that this was not the case suggests that researchers can utilize mixed C57BL/6 and DBA/2 females without increased variability compromising the statistical power of their experiments.

One other finding was of note. We found that ear notching did not affect any variable measured. This suggests there are no long-term consequences of ear notching, at least when applied with concurrent analgesia (although without analgesia this method still causes acute pain and thus constitutes a welfare issue [7], [12], [25]).

Of course, that our experiment failed to find any adverse effects of mixed strain housing does not mean that none are possible. It is possible that effects were very subtle (only detectable with larger sample sizes) or that other traits, ones we did not measure, were altered by our mixed strain paradigm. It is also possible that welfare would have been compromised, strain-typical phenotypes altered, or data rendered more variable, had the experiment gone on longer, or started at an earlier age (perhaps via cross-fostering dependent pups, cf. [65]) or had we used male subjects (cf. [66]). Finally, it is likely that not all mouse strains would cohabit in such a problem-free way (especially strains with large differences in body weight and/or temperament (see [67] for strain typical differences in male aggression). For example, in a similar experiment [66], mixing C57BL/6 with 129S mice did cause significant changes in the 129S animals’ home-cage social and feeding behaviour, and anxiety-like responses in open field tests (with anxiety-like behaviours in C57BL/6 mice also potentially modified by the mixed-strain housing too, in a manner determined by a subject’s weaning weight). Thus, it would be rash to generalize from our results to all strains/sexes/ages/etc., and more research is now needed on a range of other strains and housing/rearing conditions, as well as on male mice.

Mixed strain housing may not be appropriate for all research programs, and we do not advocate that it is adopted without further study by researchers interested in other models or variables beyond those used here. It is obviously unusable for all research involving single-housed animals (e.g. aggressive males). It is useless, like other simple marking schemes (e.g. tail marking; shaving; simple ear-notching), to anyone who needs colony level unique IDs (*c.f.* cage level unique IDs); and like these methods requires extra care that animals’ cage identities are always known. Gastrointestinal microflora typically differ between strains [68], [69], and cross-contamination would be possible in mixed strain cages - a potential confound in certain areas of research (e.g. immunology; gastroenterology). Mixed strain housing may also affect, but perhaps even render more normal, the social behaviour of mice: inbred mice have trouble distinguishing their own scent marks from those of genetically identical cage mates [70], and so mixed strain housing may facilitate more natural social behaviour and less aggression. This requires investigating, partly for its positive welfare implications, but also because it may alter results of tests reliant on social interactions. As a final caution, due to the conspicuousness of individual mice when subjects are housed like this, data collectors may need to be blind to the hypothesis (rather than the treatment, which may now be challenging), to ensure blinding.

Nevertheless, as a proof of principle and a first step in validating a refinement in laboratory mouse husbandry, this study shows that co-housing mouse strains with different coat colours can potentially be practical and safe. Specifically, researchers using female C57BL/6 and DBA/2 mice can house them together from weaning into young adulthood and still expect to replicate strain-typical results without compromising welfare. For mice housed in pairs, this practice then obviates the need for other marking techniques, with all their potential drawbacks (see Introduction), and subjectively we also found that distinguishing individuals in our mixed strain cages was far easier than relying on ear notches, as we had to for conventionally housed subjects. Therefore, in a world where group housing mice is generally both good for welfare (reviewed [71]), and sensible economically, where still we need individual-level data, and where external validity is improved by using multiple strains [27], mixed strain housing, at least for C57BL/6 and DBA/2 females, represents a new, ethically preferable, and practically and scientifically valuable way to identify individuals. There is now value in exploring other combinations of differentially-pigmented strains, especially those that are similar in aggression (see [67] for example) and body weight, so most likely to cohabit with negligible impact.
